# Joint Effect of Education and Main Lifetime Occupation on Late Life Health: A Cross-Sectional Study of Older Adults in Xiamen, China

**DOI:** 10.1371/journal.pone.0131331

**Published:** 2015-06-24

**Authors:** Manqiong Yuan, Wei Chen, Cheng-I Chu, Ya Fang

**Affiliations:** 1 State Key Laboratory of Molecular Vaccinology and Molecular Diagnostics, School of Public Health, Xiamen University, Xiamen, Fujian, China; 2 Key Laboratory of Health Technology Assessment of Fujian Province University, School of Public Health, Xiamen University, Xiamen, Fujian, China; 3 Department of Public Health, Tzu Chi University, 701, Sec. 3, Zhongyang Rd, Hualien City, Hualien County, Taiwan; "Mario Negri" Institute for Pharmacological Research, ITALY

## Abstract

**Background:**

The effects of education and occupation on health have been well documented individually, but little is known about their joint effect, especially their cumulative joint effect on late life health.

**Methods:**

We enrolled 14,292 participants aged 60+ years by multistage sampling across 173 communities in Xiamen, China, in 2013. Heath status was assessed by the ability to perform six basic activities of daily life. Education was classified in four categories: ‘Illiterate’, ‘Primary’, ‘Junior high school’ and ‘Senior high school and beyond’. Main lifetime occupation was also four categorized: ‘Employed’, ‘Farmer’, ‘Jobless’ and ‘Others’. Odds ratios (ORs) were estimated by random-intercept multilevel models regressing health status on education and main lifetime occupation with or without their interactions, adjusting by some covariates.

**Results:**

Totally, 13,880 participants had complete data, of whom 12.5% suffered from disability, and ‘Illiterate’ and ‘Farmer’ took up the greatest proportion (33.01% and 42.72%, respectively). Participants who were higher educated had better health status (ORs = 0.62, 0.46, and 0.44 for the ‘Primary’, ‘Junior high school’, and ‘Senior high school and beyond’, respectively, in comparison with ‘Illiterate’). Those who were long term jobless in early life had poorest heath (ORs = 1.88, 95% CI 1.47 to 2.40). Unexpectedly, for the farmers, the risk of poor health gradually increased in relation to higher education level (ORs = 1.26, 1.28, 1.40 and 2.24, respectively). For the ‘Employed’, similar ORs were obtained for the ‘Junior high school’ and ‘Senior high school and beyond’ educated (both ORs = 1.01). For the ‘Farmer’ and ‘Jobless’, participants who were ‘Illiterate’ and ‘Primary’ educated also showed similar ORs.

**Conclusions:**

Both education and main lifetime occupation were associated with late life health. Higher education was observed to be associated with better health, but such educational advantage was mediated by main lifetime occupation.

## Introduction

Population ageing is taking place unprecedentedly rapidly throughout the world, especially in China, the most populous country [1]. Reported by National Bureau of Statistics of the People’s Republic of China, about 212 million people aged 60+ years lived in China in 2014, accounting for 15.5% of the entire population, which was extremely higher than the ageing society threshold (10%) defined by the United Nations[2]. Moreover, due to one-child policy, China had a sharp decline of birth since 1990s, which will set it to experience much faster ageing over the coming decades. By 2050, the number of people aged 60+ years in China is estimated to exceed 300 million (http://www.gov.cn/zwgk/2013-09/13/content_2487704.htm), and it will be the country with the largest population aged 80+ years (about 90 million) [3]. As advancing in age, human sensory and motor performs decline along with various senile diseases, inexorably increasing the risk of disability [4–6]. The prevalence of disability among elderly was 2.85 times higher than among overall population [7]. Disabled people often require and occupy more health cares than their counterparts, thus putting a heavy pressure on the families as well as the society [8, 9].

Education and main lifetime occupation (MLO) in early phase of life have been frequently reported as impact factors to health of the elderly [10–16]. Many studies have found that a higher level of education is strongly associated with better health and functional status [5, 10, 12, 17]. Prior research suggested that education played a key role in building and maintaining cognitive reserves, which could be drawn upon over the life span [18]. For example, higher levels of education may promote to live with positive health behaviors [19, 20] (e.g. more frequent exercise, non-smoking, non-drinking and healthier diet) and use healthcare services to maintain good health [10]. MLO can affect the health through direct impacts (e.g. manual labor, exposure to noise and heat) and indirect impacts (e.g. income and authority) [21–23]. These impacts on health may widen in late life even beyond the working years. For example, evidence suggests that blue-collar workers were more likely to have the worst health and a high risk of disability in late life since they worked with frequent or prolonged twisting, bending, or other awkward postures during working years [24].

Despite the well-studied individual associations of education and occupation (mostly current occupation) with human health, limited studies have uncovered the complexity of their interrelationship, especially to late life health. Occupation choice is strongly associated with the educational attainment [25] and for example, individual with higher education level is more likely to be white-collar. Additionally, previous studies have revealed that current occupation and education had a joint effect on health behavior [26] and self-rated health [27]. Therefore the interaction between education and occupation should not be ignored in analyses of human health. Moreover, because the effects of education and occupation on health are cumulative over the life span, they may widen in late life health. In light of this, using MLO instead of current occupation or latest occupation can more effectively tease out the cumulative effect of occupation on late life health.

The current study aimed to reveal interrelationships between education and MLO to the late life health based on a large scale cross-sectional survey among the elderly in China. We first summarized the characteristic of the participants by health status. Then random-intercept multilevel logistic regression analyses were performed with and without accounting for the interactions, adjusting by the covariates. Odds ratios (OR) of disability were mainly used to indicate the effects of impact factors on the late life health.

## Materials and Methods

### Study Population

A cross-sectional survey was performed across 173 communities in Xiamen, China, in 2013. We enrolled 14,292 participants aged 60+ years who were local household registered by multi-stage stratified sampling procedure. At the time of this study, a total of 261,043 individuals were aged 60+ years in Xiamen and therefore about 5.5% of overall elderly populations were covered. Information of basic demographic characteristics, life habits and health status was completed by face-to-face interview, each taking 15 to 20 minutes without any gifts or rewards.

### Ethics Statement

Ethical review of this study was approved by the Committee of School of Public Health, Xiamen University. Written informed consent was obtained from each participant at the first page of the questionnaire. Interviewers read the questions exactly as they appear on the survey questionnaire. The choices of answers to the questions were provided verbally by the participants and then interviewers wrote the response code on the questionnaire.

### Health Status Assessment

In the present study, the primary outcome of interest was the health status, which was measured by Katz Activities of Daily Living Scale[28]. In this scale, the set of activities assessed were dressing, feeding, transferring (getting in/out of bed), walking (walking around inside), bathing and using the toilet. Participants were asked to indicate whether they were capable of doing these activities independently. Three response options (*completely independent*, *need some helps*, and *completely dependent on others*) were provided. Health status was classified into five categories: (1) participants were classified as totally independent when they reported that they were able to perform all the six activities completely independent; (2) if needing some helps on one or more of these activities but not reporting any ‘*completely dependent on others*’, they were classified as relatively independent; (3) participants had a mild disability if they were completely dependent on others to perform one or two of these activities; (4) moderate disability was classified when such inabilities occurred in three or four activities; and (5) most severely, participants were considered as total disability if they were unable to perform five or more of these activities. Furthermore, we dichotomized the health status into non-disability and disability in our regression analyses. The participants who were totally independent composed the non-disability group, and the disability group consisted of the other four health statuses.

### Impact Factors

The exposures of interest were education and MLO. The education was classified into four categories: ‘Illiterate (no formal education)’, ‘Primary (less than 6 years formal education)’, ‘Junior high school (6-8years formal education)’ and ‘Senior high school and beyond (9 years or more formal education)’. The MLO also contained four categories including ‘Employed (government, NGO, and private company)’, ‘Farmer (farming, fishing, and forestry)’, ‘Jobless (housewife and homemaker)’ and ‘Others (merchant, temporary worker, commercial household, and others)’. Demographic characteristics included sex and residence (urban or rural). Early life health behaviors included smoking history (never, sometimes, often, or quit), dietary habit (salt-light, salt-medium, or salt-heavy), alcohol drinking history (never, sometimes, often, or quit), exercise practicing (never, sometimes, or often) and sleep quality (very bad, bad, fair, good or very good). Besides, number of chronic disease was also included as a covariate. Particularly, as age played a major role in health of the elderly, we explored the effects of both exposures under different age levels and categorized the age in five-year groups (60–64, 65–69, 70–74, 75–79, 80–84, and 85 years and older).

### Statistical analysis

Descriptive statistics were used to summarize the characteristics of the participants by the binary health status. For more intuitively, we illustrated the distribution of age by the five categorized health statuses and the two exposures (education and MLO) using violin plot with jittering points. The interspersion of participants jointly by education and MLO was shown by a bubble plot, in which the association between education and MLO can be intuitively presented. Second, since the survey was based on a multi-stage stratified cluster sampling, nine multilevel logistic regressions were performed where the 173 communities were considered as one level of hierarchy. We modeled the binary health status on age only, education only, MLO only, age and education, age and MLO, and all variables with random intercept for community to account for clustering within community, adjusting for the abovementioned covariates (models 1–6). Finally, interactions among age and the two exposures were added (models 7–9). ORs were estimated using the function for linear combinations of coefficients, where the age ‘60–64’, education ‘Illiterate’ and MLO ‘Employed’ were the reference categories, respectively. All the figure and analyses were completed in R. More specifically, the multilevel logistic regressions were performed using the function of *glmer()* in the package of ‘*lme4’* and the ORs when accounted for interactions were estimated by the function of *glht()* in the package of ‘*multcomp’*.

The nine regression models with random intercept for community were expressed as follows:

Model 1: logit (Health status) ~ Age + Covariates;
Model 2: logit (Health status) ~ Education + Covariates;
Model 3: logit (Health status) ~ MLO + Covariates;
Model 4: logit (Health status) ~ Age + Education + Covariates;
Model 5: logit (Health status) ~ Age +MLO + Covariates;
Model 6: logit (Health status) ~ Age + Education + MLO + Covariates;
Model 7: logit (Health status) ~ Age + Education + Age×Education + Covariates;
Model 8: logit (Health status) ~ Age + MLO +Age×MLO + Covariates;
Model 9: logit (Health status) ~ Age+ Education + MLO + Education×MLO + Covariates.

## Result

### Descriptive analyses

Among the 14,292 valid questionnaires, 13,880 (97.1%) had complete data on all variables mentioned above and were included in the analyses. Table 1 presented participant characteristics by binary health status. The mean age was obviously older for the disabilities than non-disabilities (78.69 vs. 70.46 years). The participant who was female, lower educated, long term jobless, suffered from more kinds of chronic disease, and lived in rural was more likely to suffer from a disability. Unexpectedly, most participants with disabilities reported they had never smoked, drank alcohol or practiced exercises. Those whose sleep quality was reported good or very good showed lower prevalence of disability.

**Table 1:** Basic characteristic of 13,880 participants according to the health status.

Characteristic	Non-disability	Disability	Disability Prevalence (%)	95% CI^a^ of prevalence
**Total, N**	12145	1735	12.50	11.95–13.05
**Age, mean(SD) /years**	70.46(7.71)	78.69(8.93)	-	-
**Sex, N(%)**				
Female	6163(50.75)	1022(58.90)	14.22	13.41–15.03
Male	5982(49.25)	713(41.10)	10.65	9.91–11.39
**Education, N(%)**				
Illiterate	3647(30.03)	936(53.95)	20.42	19.25–21.59
Primary	3842(31.63)	461(26.57)	10.71	9.79–11.63
Junior high school	2415(19.88)	184(10.61)	7.08	6.09–8.07
Senior high school and beyond	2241(18.45)	154(8.88)	6.43	5.45–7.41
**MLO, N(%)**				
Employed	3379(27.82)	283(16.31)	7.73	6.86–8.6
Farmer	5035(41.46)	895(51.59)	15.09	14.18–16
Jobless	1458(12.00)	315(18.16)	17.77	15.99–19.55
Others	2273(18.72)	242(13.95)	9.62	8.47–10.77
**Residence, N(%)**				
Urban	6079(50.05)	657(37.87)	9.75	9.04–10.46
Rural	6066(49.95)	1078(62.13)	15.09	14.26–15.92
**Number of Chronic disease, N(%)**				
0	4852(39.95)	232(13.37)	4.56	3.99–5.13
1	3707(30.52)	475(27.38)	11.36	10.40–12.32
2	2167(17.84)	500(28.82)	18.75	17.27–20.23
> = 3	1419 (11.68)	528(30.43)	27.12	25.15–29.09
**Smoking history**				
Never	7640(62.91)	1194(68.82)	13.52	12.81–14.23
Sometimes	1663(13.69)	224(12.91)	11.87	10.41–13.33
Often	2167(17.84)	124(7.15)	5.41	4.48–6.34
Quit	675(5.56)	193(11.12)	22.24	19.47–25.01
**Dietary habit** ^b^				
Light	5404(44.50)	889(51.24)	14.13	13.27–14.99
Medium	5802(47.77)	689(39.71)	10.61	9.86–11.36
Heavy	939(7.73)	157(9.05)	14.32	12.25–16.39
**Alcohol Drinking**				
Never	8038(66.18)	1337(77.06)	14.26	13.55–14.97
Sometimes	3018(24.85)	202(11.64)	6.27	5.43–7.11
Often	687(5.66)	27(1.56)	3.78	2.38–5.18
Quit	402(3.31)	169(9.74)	29.60	25.86–33.34
**Exercise**				
Never	3890(32.03)	1249(71.99)	24.30	23.13–25.47
Sometimes	4755(39.15)	411(23.69)	7.96	7.22–8.7
Often	3500(28.82)	75(4.32)	2.10	1.63–2.57
**Sleep Quality** ^c^				
Very bad	192(1.58)	160(9.22)	45.45	40.25–50.65
Bad	1270(10.46)	498(28.7)	28.17	26.07–30.27
Fair	6662(54.85)	853(49.16)	11.35	10.63–12.07
Good	3161(26.03)	173(9.97)	5.19	4.44–5.94
Very good	860(7.08)	51(2.94)	5.60	4.11–7.09

^a^ confidence interval.

^b^ salt-light: salt intake<6g/day, salt-medium: salt intake 6-18g/day, salt heavy: salt intake >18g/day.

^c^ Sleep quality was self-rated.

**Fig 1:**
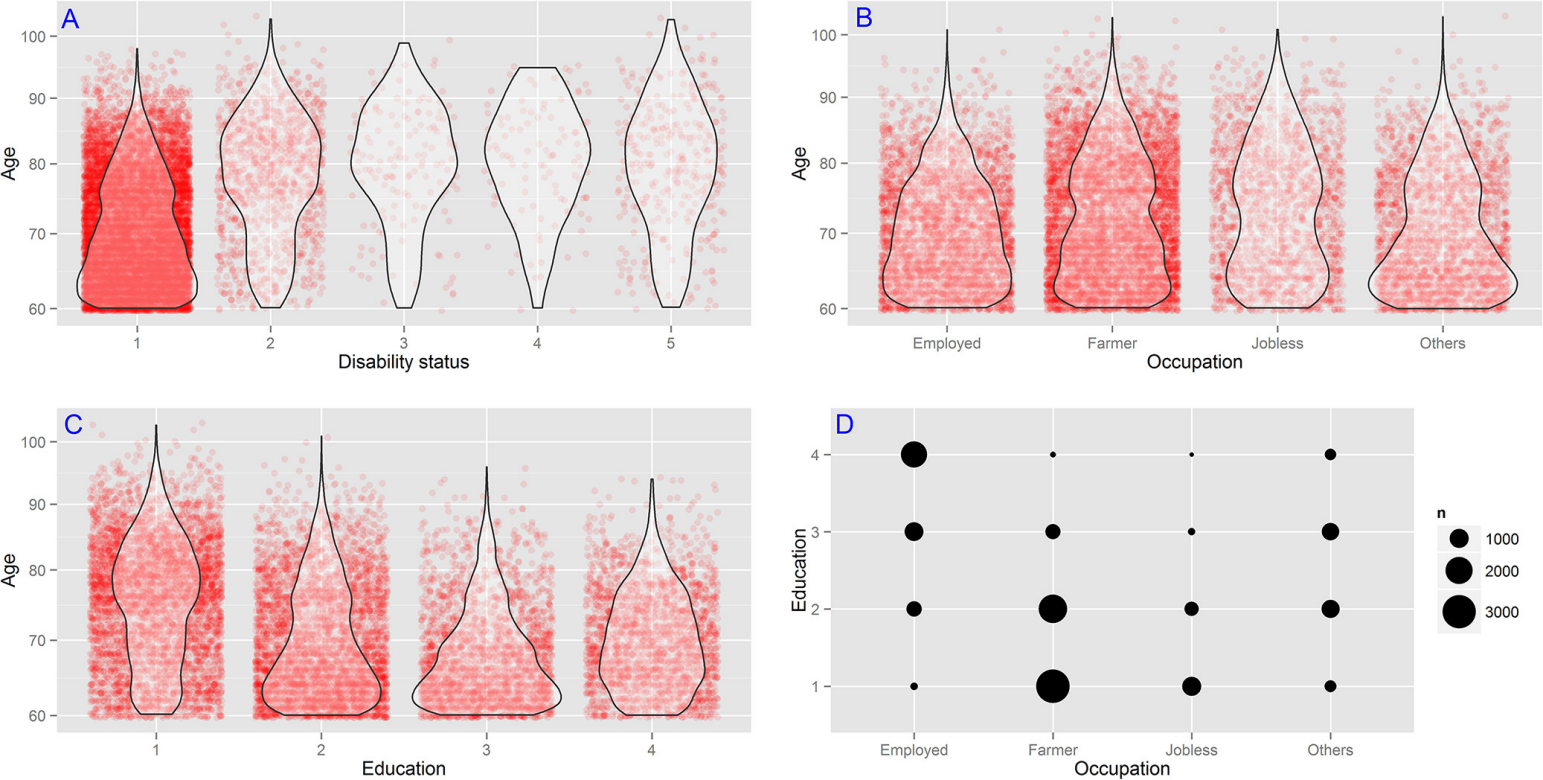
Violin plots with jittering points (A, B, C) of participants’ age under health status, education and MLO, and bubble plot (D) for interspersion of participants by education and MLO. The number of jittering points (A, B, C) was the number of participants and the violin curves were rotated kernel density curve to display the probability density of age. The bubble size in D reflected the number of participants. For health status, 1 = totally independent, 2 = relatively independent, 3 = mild disability, 4 = moderate disability and 5 = total disability. For education, 1 = Illiterate, 2 = Primary, 3 = Junior high school and 4 = Senior high school and beyond.

Fig 1 depicts the participant distributions among age, five categorized health status, education and MLO. The **A** presented the age distribution among five health statuses by violin plot with jittering points. We noticed a decremented trend in number of participants and an upward central tendency of age when the health status went poorer. The violin plots for the disability group (the right four) presented a similar central tendency around 80 years while the non-disability group showed obviously lower one around 63 years. Quantitatively, only 14.7% of the totally independent participants were aged 80 years or over while such proportions were much higher for the other groups which were 47.52%, 54.48%, 58.49% and 52.55%, respectively for the relatively independent, mild disability, moderate disability and total disability. The **B** reflected the age distributions among four kinds of MLO. ‘Farmer’ took the largest proportion of participants as it had the most concentrated jittering points. Quantitatively, the mean ages were oldest for ‘Jobless’ followed by ‘Farmer’, ‘Employed’ and ‘Others’, which were 73.36, 72.14, 70.45 and 70.15 years old respectively. Moreover, ‘Employed’ had a lowest proportion (13.05%) of senior elderly aged 80 years or over, while such proportion was more than doubled (27.07%) for ‘Jobless’. The distributions of age among education were illustrated in **C**. A downward central tendency and top tail of age were showed as educational attainment advance. More concretely, the elderly who did not receive any formal education (Illiterate) were concentrated at senior age. For ‘Primary’ and ‘Junior high school’ violin plots, the central tendency located at a younger age around 63 years. Such obvious younger trend, however, stopped at ‘Senior high school and above’. Quantitatively, the proportion of senior elderly aged 80 years or over for the ‘Illiterate’ (35.57%) was highest, followed by the ‘Primary’ (14.18%), the ‘Senior high school and beyond’ (9.52%) and the ‘Junior high school’ (7.16%). Finally, the number of participants across different educational attainments and MLO was represented by the bubble size in **D**. Regarding to the MLO, participants who received more educations took larger proportion among the ‘Employed’, however such trend was converse among the ‘Farmers’ and ‘Jobless’. In terms of education, those who had higher level of education were more likely to be ‘Employed’, and those with lower education were more likely to be ‘Farmer’.

### Multilevel logistic regressions without interaction

Table 2 depicted the odds ratio of disability across different levels of age, education and MLO. Expectedly, increased odds were observed as age advanced in all the models which included age as a predictor (models 1, 4, 5 and 6). There was a decreasing trend of odds as educational attainment improved when the MLO was not entered the models (models 2 and 4). Yet such trend was obliterated when MLO entered as a predictor (model 6). The elderly who were long term ‘Jobless’ in early life had the highest risk of disability, followed by ‘Farmers’, ‘Employed’ and ‘Others’ (models 3, 5 and 6). Such trend was consistent in all the models no matter whether the age and education entered the model or not.

**Table 2:** Odds ratios (95% confidence intervals) obtained from multivariable adjusted^a^ multilevel logistic models (model 1–6).

	Model1	Model2	Model3	Model4	Model5	Model6
**Age, years**						
60~	**1.00**			**1.00**	**1.00**	**1.00**
65~	1.39(1.08, 1.79)			1.40(1.09, 1.81)	1.38(1.07, 1.78)	1.39(1.08, 1.79)
70~	1.72(1.33, 2.22)			1.73(1.34, 2.24)	1.71(1.33, 2.21)	1.73(1.34, 2.24)
75~	2.97(2.34, 3.76)			2.96(2.33, 3.78)	2.92(2.30, 3.70)	2.95(2.32, 3.77)
80~	4.71(3.70, 6.00)			4.67(3.64, 6.00)	4.62(3.63, 5.89)	4.67(3.64, 5.99)
85 or above	12.12(9.44, 15.55)			11.96(9.24, 15.49)	11.73(9.13, 15.07)	11.84(9.14, 15.34)
**Education**						
Illiterate		**1.00**		**1.00**		**1.00**
Primary		0.62(0.52, 0.72)		0.99(0.83, 1.18)		1.03(0.86, 1.23)
Junior high school		0.46(0.37, 0.58)		0.96(0.75, 1.23)		1.03(0.80, 1.33)
Senior high school and beyond		0.44(0.34, 0.57)		0.82(0.63, 1.08)		0.88(0.65, 1.20)
**MLO** ^**b**^						
Employed			**1.00**		**1.00**	**1.00**
Farmer			1.36(1.05, 1.77)		1.14(0.87, 1.5)	1.1(0.82, 1.47)
Jobless			1.88(1.47, 2.40)		1.49(1.15, 1.93)	1.43(1.08, 1.90)
Others			0.92(0.73, 1.15)		0.90(0.71, 1.14)	0.86(0.67, 1.11)

^a^ All the six models used the same set of covariates: sex, residence, smoking history, dietary habit, alcohol drinking history, exercise practicing, sleep quality and number of chronic disease.

^b^MLO: main lifetime occupation

### Multilevel logistic regressions with interactions

Table 3 depicts the ORs and corresponding 95% confidence intervals (CI) for the joint effects of age with education, and age with MLO, on health status. The group of ‘Illiterate’ aged 60–64 years was the reference (model 7). An obvious increase trend of ORs was presented as age advanced. Higher level of education showed slightly lower ORs among senior elderly aged 85 years or over. Yet such educational advantage was inconspicuous for the other age groups. The elderly who was senior high school and beyond educated always showed to have lowest odds of disability, except for the ‘75~’ and ‘80~’ age groups. For the association of age and MLO, the group of ‘Employed’ aged 60–64 years was the reference (model 8). Also a notable increase trend of ORs was showed as age advanced. The elderly who were long term jobless showed to have greatest odds of disability in almost all age group. ‘Others’ had lowest odds of disability among the elderly aged 80 years or older and younger than 70 years. For ‘70~’ and ‘75~’ age groups, ‘Employed’ had lowest odds of disability. When we included the interaction of education and MLO in the model (model 9), the OR pattern was much more complex (Table 4). The elderly who had ‘junior high school’ education and were long term jobless presented the highest odds of disability, followed by the farmers with senior high school and beyond education. Unexpectedly, among the farmers, the elderly with higher level of education, however, had higher odds of disability, which was converse to the general trend and also to the result obtained before. Illiterate and primary educated elderly had almost the same odds of disability among ‘Farmers’ and ‘Jobless’ participants. The ‘Junior high school’ educated had similar odds of disability to the ‘Senior high school and beyond’ educated for ‘Employed’ participants. Accordingly, we may conclude the MLO has a more robust effect on late life health than education, and the effect of education will be obliterated by MLO.

**Table 3:** Odds ratios (95% confidence intervals) obtained from multivariable adjusted multilevel logistic models regressing health status on age and education (model 7), and age and MLO (model 8) accounting for their interactions.

	Age (years)					
	60~	65~	70~	75~	80~	85 or above
**Education attainment**						
Illiterate	1.00	1.12(0.70,1.81)	1.52(0.97,2.39)	2.57(1.71,3.87)	4.12(2.75,6.18)	10.95(7.30,16.41)
Primary	0.82(0.52,1.31)	1.18(0.74,1.88)	1.77(1.1,2.83)	2.56(1.61,4.05)	4.04(2.51,6.49)	10.46(6.32,17.32)
Junior high school	0.93(0.54,1.58)	1.56(0.95,2.58)	1.28(0.71,2.30)	1.83(1.00,3.35)	3.62(1.82,7.20)	9.69(4.51,20.82)
Senior and beyond	0.52(0.22,1.19)	0.89(0.49,1.63)	1.08(0.60,1.96)	3.00(1.75,5.12)	3.91(2.11,7.24)	5.86(2.56,13.39)
**MLO** ^**a**^						
Employed	1.00	1.46(0.81,2.66)	1.79(1.02,3.16)	3.12(1.80,5.41)	5.93(3.38,10.42)	13.24(7.05,24.83)
Farmer	1.37(0.78,2.4)	1.85(1.06,3.21)	2.29(1.32,3.97)	3.53(2.07,6.05)	5.25(3.07,8.98)	14.61(8.51,25.08)
Jobless	1.63(0.81,3.26)	2.43(1.28,4.59)	2.19(1.08,4.45)	4.40(2.45,7.89)	8.51(4.76,15.21)	20.15(11.35,35.77)
Others	0.85(0.44,1.66)	1.10(0.58,2.08)	1.83(0.97,3.45)	3.66(2.07,6.50)	4.60(2.54,8.32)	10.86(5.79,20.36)

^a^MLO: main lifetime occupation

**Table 4:** Odds ratios (95% confidence intervals) obtained from multivariable adjusted multilevel logistic models regressing health status on education and MLO (model 9), accounting for interactions of education and MLO.

MLO^a^	Education			
	Illiterate	Primary	Junior high school	Senior high school and beyond
**Employed**	1.00	1.59(0.84, 3.02)	1.01(0.53, 1.92)	1.01(0.54, 1.86)
**Farmer**	1.26(0.69, 2.31)	1.28(0.69, 2.4)	1.40(0.69, 2.84)	2.24(0.77, 6.47)
**Jobless**	1.62(0.88, 2.97)	1.62(0.84, 3.13)	3.01(1.33, 6.8)	1.11(0.32, 3.9)
**Others**	1.2(0.63, 2.29)	0.91(0.48, 1.7)	1.06(0.54, 2.06)	0.89(0.42, 1.9)

^a^MLO: main lifetime occupation

## Discussions

The findings in this study showed that higher education was associated with better health, but such educational advantage may be mediated by MLO. With or without accounting for age and education, the elderly who were long term jobless in early life were always showed to have highest odds of disability, followed by farmers. In contrast, the ‘Employed’ participants had a relatively low risk of disability.

Although educational level played a particular role in the choice of MLO, our result suggested its effect on late life health was disordered when accounting for MLO. There are several possible explanations to this finding: (1) the highest level of education is achieved at young adulthood for most people while the occupation usually starts at the end of the educational process [29]. To late life health, the effect of occupation may take priority over the effect of education since it happened much more recently to the elderly. (2) The cumulative effect of occupation is stronger because its duration is significantly longer than education. In general, people begin formal school at about 6 years old and finish primary school at 12 years old, junior high school at 15 years old, and senior high school at 18 years old. Therefore, the education duration is usually less than 12 years. Nevertheless, the retirement age is 55 years old for female and 60 years for male in China and most people keep on working until retirement. So conservatively, the duration of occupation is usually longer than 30 years, more than doubled of the educational duration. (3) Occupation choice is a channel for education to affect the health [30]. If the channel is damaged or disordered, the effect of education may be obliterated. For example, educated individuals can avoid physically demanding jobs and this reduces the risk of disability [31]. However, if the educated individuals engaged in manual labor, the positive effect of education might probably be covered by the negative effect of occupation. (4) High education was achieved first, before the health was affected by some severe diseases, and such unhealthy changes may affect the occupation choice later in life. In light of this situation, the effect of education on the health of the elderly could be misleadingly explained by the occupation but was actually affected by the unhealthy changes.

Beyond our expectation, higher education level showed higher odds of disability among farmers. Although such finding had never been published before, some explanations could be: (1) In countryside, farmers who received more education were more likely to engage in some technical works, while the low educated farmers were relatively conservative and therefore they were more likely to be general laborers. For example, the low educated farmers may plough the land by buffalos while the high educated were more likely to use tractors. Likewise, in countryside house construction teams, the user of electric equipment such as stirring machine often received more education while most of those who shouldered the slurry were illiterates or primary educated. Moreover, those technical works generally had higher risks of physical inactivity. Therefore high educated farmers were more likely to suffer from a disability, especially for senior high school and beyond educated farmers as shown in our result. Additionally, the illiterate and primary educated both tended to be general laborers and they may not show obvious differences in work choice among farmers, so they had similar low odds of disability. (2) Education and occupation were two important indicators of social-economic status [32]. In China, lower educated and farmers were generally considered as lower social class. Therefore high educated individuals were less likely to be farmers and, in turn, those high educated farmers were more likely to feel unsatisfied and complain their lives. The cumulative negative mental attitudes ultimately lead to physical health problem in late life. Altogether, the evidence was scant on why high educated farmers had high risk of disability. Longitudinal studies are needed to detect or verify the causations to this result and the occupation change history can provide important messages.

With or without interaction, elderly who were long term jobless in early life always showed relatively high odds of disability among all age levels. This result was in line with literatures [13, 21, 33, 34] and the relationship between jobless and poor health, both for mental and physical, had been well documented [34–37]. Firstly, the jobless were more likely to suffer from impaired mental health including depression, ashamed, anxiety, and stress, and they were usually less self-confident and self-esteemed [33]. Additionally, the impaired mental health can, in turn, lead to poor health habits like excess drinking, smoking, lack of exercise, and a sedentary lifestyle [38, 39]. Secondly, the jobless were more likely to delay in seeking health care service due to cost [37]. Therefore, the status of jobless should not only be considered a minor inconvenience but also a risk factor of health. Moreover, the jobless should be counseled to pay more attention to better health habits.

Occupation and education are two core indicators of social-economic status. They are strongly related, but not interchangeable[40]. Using more than one indicators of social-economic status can make considerable gains in understanding the social-economic health disparities[41]. However, in many studies, only one of them has been considered to detect the social-economic health disparities. In cases where both are included, they just focus on the effect of one indicator on health outcomes after adjusting for the other one. In the present study, we not only included both of them, but also considered their joint effect on late life health, which can provide more clues in understanding the social-economic health disparities. Moreover, reduction of social-economic health disparities is an important goal of public health[42], and our results should be helpful for health policy makers to optimize their strategies.

This study has a number of strengths, including the use of the recent large scale and representative samples among the elderly. Moreover, it offered a new perspective on the importance of joint effect between education and MLO on the late life health. Furthermore, we used MLO instead of current occupation or latest occupation, which can more effectively tease out the cumulative effect on late life health. Nevertheless, there were also some limitations with the present study. First, the data in our study were from a cross-sectional survey and this limited the interpretation of our results. Meanwhile, the participants were sampled from one city in China, which may be local characterized and in turn exist some bias for interpretation in countrywide. Second, the changes of some covariates in life span, such as life behaviors, cannot be accounted for due to the lack of this information, which will be concerned in our follow-up survey. Third, the participants in our study were born between 1910 and 1954. In the past few decades, some huge historic events (e.g. Foundation of the People’s Republic of China, The Cultural Revolution, and Reform & Opening) set China experience tremendous change involving both education and occupation. The effect of historical impress cannot be singled out and thus the results may be limited to the elderly born in that era.
